# Somatosensory Training Improves Proprioception and Untrained Motor Function in Parkinson's Disease

**DOI:** 10.3389/fneur.2018.01053

**Published:** 2018-12-10

**Authors:** Naveen Elangovan, Paul J. Tuite, Jürgen Konczak

**Affiliations:** ^1^School of Kinesiology, University of Minnesota, Minneapolis, MN, United States; ^2^Department of Neurology, University of Minnesota, Minneapolis, MN, United States

**Keywords:** proprioceptive learning, somatosensory learning, sensorimotor learning, robotic rehabilitation, neurorehabilitation, movement disorders

## Abstract

**Background:** Proprioceptive impairment is a common feature of Parkinson's disease (PD). Proprioceptive function is only partially restored with anti-parkinsonian medication or deep brain stimulation. Behavioral exercises focusing on somatosensation have been promoted to overcome this therapeutic gap. However, conclusive evidence on the effectiveness of such somatosensory-focused behavioral training for improving somatosensory function is lacking. Moreover, it is unclear, if such training has any effect on motor performance in PD.

**Objective:** To investigate, whether proprioception improves with a somatosensory focused, robot-aided training in people with PD (PWPs), and whether enhanced proprioception translates to improved motor performance.

**Method:** Thirteen PWPs of mild-moderate clinical severity were assessed and trained ON medication using a robotic wrist exoskeleton. Thirteen healthy elderly participants served as controls. Training involved making increasingly accurate, continuous, precise small amplitude wrist flexion/extension movements. Wrist position sense acuity, as a marker of proprioception function, and spatial error during wrist pointing, as a marker of untrained motor performance, were recorded twice before and once after training. Functional hand writing kinematics exhibited during training were evaluated in the PD group for determining training-induced changes.

**Results:** Training improved position sense acuity in all PWPs (mean change: 28%; *p* < 0.001) and healthy controls (mean change: 23%; *p* < 0.01). Second, 10/13 PD participants and 10/13 healthy control participants had reduced spatial movement error in the untrained wrist pointing task after training. Third, spatial error for the functional handwriting tasks (line tracing and tracking) did not improve with training in the PD group.

**Conclusion:** Proprioceptive function in mild to moderate PD is trainable and improves with a somatosensory-focused motor training. Learning showed a local transfer within the trained joint degree-of-freedom as improved spatial accuracy in an unpracticed motor task. No learning gains were observed for the untrained functional handwriting task, indicating that training may be specific to the trained joint degree-of-freedom.

## Introduction

Parkinson's disease (PD) is associated with somatosensory abnormalities that include impaired haptic (1, 2) and tactile perception (3–5), altered thermal and mechanical pain perception (6), and decreased proprioceptive function [for reviews: (7–9)]. Features of altered proprioception in PD include increased active and passive joint position sense errors (10, 11), and elevated detection thresholds for position (12) and passive motion sense (13). Neurophysiological correlates of abnormal somatosensory function in PD are altered long latency somatosensory evoked potentials to median nerve stimulation (14), reduced activation of the contralateral sensorimotor cortex and supplementary motor areas during haptic object discrimination (2), and reduced intra-cortical inhibition to changes in wrist position (15).

Anti-parkinsonian medication and deep brain stimulation (DBS) only partially restore proprioceptive and haptic acuity (13, 16) in people with PD (PWPs). PWPs show about a 15% increase in haptic sensitivity during their ON medication state (16), while deep brain stimulation of the subthalamic nucleus DBS improved the haptic discrimination threshold by 26% (17), indicating that pharmacological and neuromodulation interventions for PD are not entirely successful in restoring proprioceptive function comparable to healthy adults.

This opens a potential avenue for somatosensory-based interventions as add-on therapies for PD. There has been great interest in this approach because of the potential benefits on motor function associated with somatosensory training. Indeed several forms of proprioceptive or somatosensory-based training have been proposed. They typically seek to improve motor function by focusing on proprioceptive and/or tactile afferent signals. A recent systematic review identified that proprioceptive training improves measures of proprioceptive function in healthy adults by about 26% (18). Such training induces concurrent processes of proprioceptive and motor learning, leads to short-term neuroplastic changes (19), which ultimately enhances motor performance (20–22). Stroke patients participating in similar forms of proprioceptive training revealed altered cortical activity in somatosensory and sensorimotor processing areas (23), which resulted in improved movement accuracy indicating that sensory learning transfers to motor function (24).

Evidence for effectiveness of such training in PD is mixed. Somatosensory stimulation training in the form of whole body vibration produced inconsistent effects on postural control in PWPs (25–27). At present, a sensory training with focus on upper limb proprioception has never been evaluated in PD. Moreover, there are no data available to indicate whether and to what extent proprioceptive function can be restored through somatosensory training in PD. In addition, it remains unproven whether training-induced improvement in somatosensory function in PD translates to improved motor control. Finally, there has been limited to no evidence if learning effects transfer or generalize to other functional tasks. In summary, it is important to establish if sensory-based training improves PWPs. Thus, the purpose of this study was to examine systematically the effectiveness of a somatosensory-based training intervention on sensory and motor function in PWPs. The focus of the training was wrist/hand function. Specifically, we examined, if it improved (1) proprioceptive acuity of the wrist joint, (2) motor performance of an untrained wrist pointing task (evidence of local transfer), and (3) performance in a multiple degree-of-freedom handwriting task (global or functional task transfer).

## Methods

### Participants

This open-label pilot study was approved by the University of Minnesota Human Research Protection Program. The procedures followed were in accordance with the Minnesota Human Research Protection Program and the World Medical Association Declaration of Helsinki. All participants gave written informed consent before enrollment. Thirteen individuals with idiopathic PD (Age, *mean* ± *sd*: 61.7 ± 6.8 years) were recruited for the PD group and enrolled from the University of Minnesota's Movement Disorders Center (Table 1). Inclusion criteria were: (1) modified Hoehn and Yahr stage of 3 or below (ON meds), (2) Mini Mental Status Exam (MMSE) score of 24 or above, 3) aged between 35 and 75 years, (4) able to walk, (5) no to moderate arm rigidity (ON meds), (6) no to moderate resting arm tremor (ON meds), (7) no history of motor fluctuations, (8) no history of levodopa-induced dyskinesia, (9) no action tremor (ON meds), (10) no history of musculoskeletal/neurological disorders other than PD, and were on stable medication regimens for at least 1 month before enrollment. Thirteen healthy elderly adults (Age, *mean* ± *sd*: 67.0 ± 6.5 years; 8 females and 5 males) with no known neurological conditions were recruited to serve as the healthy control group. All the participants in the healthy control group were right-handed with a mean handedness score of 76.5 ± 29.0. As a group the healthy older adult control group was significantly older than the PD cohort.

**Table 1:** Research participant demographics and clinical characteristics.

**ID**	**Age (in years)**	**Gender**	**Disease duration (in years)**	**UPDRS III (Motor evaluation)**	**MMSE score**	**Most affected side**	**Dominant side**	**Levodopa equivalent dosage (in mg) (28)**
1	65	F	5.0	26	28	Left	Right	300
2	55	M	3.4	13	29	Right	Right	300
3	66	M	2.9	10	25	Right	Left	300
4	49	F	0.8	5	30	Left	Left	200
5	74	M	0.4	36	28	Right	Right	0
6	54	F	0.9	16	29	Right	Right	0
7	61	M	10.0	28	26	Left	Right	550
8	64	F	2.3	9	30	Right	Right	225
9	67	F	2.4	11	29	Right	Right	300
10	67	F	2.3	8	30	Right	Right	300
11	55	F	5.4	9	28	Right	Right	800
12	63	F	3.3	8	29	Right	Right	300
13	62	M	2.9	13	30	Left	Right	450

*UPDRS refers to Unified Parkinson's Disease Rating Scale. Motor evaluation section scores are reported here. MMSE refers to Mini Mental Status Exam*.

### Research Design

The study employed two groups, PD group and healthy elderly control group, single treatment pre/post-test design. PD participants were tested “on” medication and visited the lab twice within 7 days (Table 2). On Day 1, all PWPs were rated by N.E. using the motor examination sub section of the Unified Parkinson's Disease Rating Scale (UPDRS), and had cognitive functions assessed using MMSE. Subsequently, participants' proprioceptive acuity and motor performance was assessed. On Day 2, participants were evaluated for proprioceptive acuity, motor performance, training performance, and handwriting, both before and after proprioceptive training. Double baseline measurements of proprioceptive acuity and motor performance on Day 1 and Day 2 before training served a two-fold purpose: (1) to evaluate reliability of our measurements and (2) to serve as their own controls. In a single session, healthy controls were tested for proprioceptive acuity and motor performance, both before and after proprioceptive training. While the PD group had a double baseline evaluation for the proprioceptive and motor performance, the healthy control group were evaluated once, as the reliability on healthy controls has been established elsewhere (29).

**Table 2:** List and evaluation time points of outcome measures.

	**Day 1**	Day 2^*^	
	**Baseline 1**	**Baseline 2**	**After Training**
UPDRS	✓		
MMSE	✓		
Proprioceptive Evaluation	✓	✓	✓
Motor Evaluation	✓	✓	✓
Handwriting Evaluation		✓	✓

* *Day 2 evaluation occurred within 1–7 days of Day 1 evaluation*.

*UPDRS refers to Unified Parkinson's Disease Rating Scale. MMSE refers to Mini Mental Status Exam. All participants underwent double baseline evaluations for proprioceptive function and motor evaluation*.

### Wrist Robotic Device

**Figure 1:**
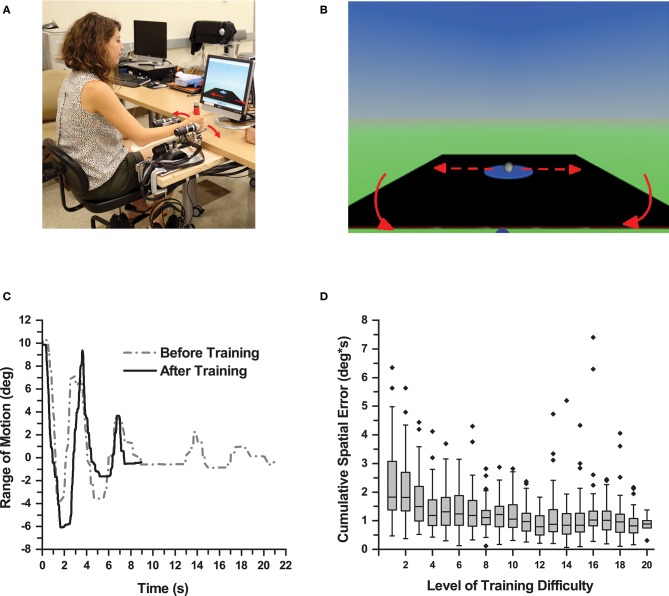
Proprioceptive Training. **(A)** Participant performing a training trial using the WristBot (Media consent provided by the participant for publication). **(B)** Monitor screenshot displaying the virtual table and the virtual ball. The participant wrist movement caused the table to tilt resulting in displacement of the ball. **(C)** Wrist displacement of one participant during a trial before and after training. After training, the participant produced larger displacement and shorter duration to complete a trial in this balancing task. **(D)** Box plot showing cumulative spatial error of all participants across all levels of training difficulty. As participants achieved higher levels of training difficulty, the cumulative spatial error in each level decreased for the all participants demonstrating motor learning.

The *WristBot* (Figure 1A) is a three degree-of-freedom robotic exoskeleton, that allows for full range-of-motion wrist flexion/extension, ad/abduction, and forearm supination/pronation (30). This haptic robot is a fully backdriveable system with capability to deliver precise haptic, joint position, and velocity stimuli. Optical encoders record wrist and forearm position at 200 Hz with a spatial resolution of 0.0075°. The robot is coupled to a virtual reality environment that provide visual feedback to the user about his/her wrist position. Validity and reliability of this robot-based proprioceptive assessment were established previously (29).

### Training Procedure

Participants sat on a height-adjustable chair with their more-affected forearm on the WristBot's arm rest and observed a virtual ball rolling on a tiltable table (Figure 1B). In each trial, participants had to move the ball to a target zone by making precise, small amplitude back, and forth wrist flexion/extension movements and, once in the zone, to hold the position for 5 s. A training trial was *successful*, if the ball reached the target zone within 60 s. After completing three successful trials at a particular level of difficulty, the software automatically moved to the next higher level. Within a given level of difficulty, the horizontal, neutral position of the table (where the ball would not move) was altered. Initially it was set to 10° wrist flexion and then changed to 15° and 20° when a trial was completed successfully. The level of difficulty increased by altering the mechanical properties of the virtual environment (e.g., mass, velocity gain, and dampening force of the ball, gravitational constant). These changes increased the ball's responsiveness to table motion, which the learner had to control through his/her wrist motion. Thus, as learners advanced to higher levels of difficulty, they had to make increasingly accurate wrist movements to control the virtual ball (Figure 1D). Each participant completed 60 trials, which took participants 13–53 min to complete (mean training duration: 30 min). They could take a 5-min break after 30 trials. This setup provided participants with the opportunity to be successful at their personal level, which implied they could achieve different levels of difficulty by the end of training.

### Assessment and Measurements of Sensorimotor Learning and Motor Transfer

#### Evaluation of Training Task-Specific Motor Learning

To evaluate the direct effects of training, participants in the PD group completed 3 trials of the training task at a difficulty level of 15, both before and after training. Based on the wrist angle-time series data, the instantaneous lateral deviation (LD) from the neutral (balanced) position was computed and a cumulative spatial error determined for each trial following:

(1) $$\begin{array}{l} CSE_{trial}=\int_{i=1}^{n}\sum |\;LD_{i}\;|\;dt \end{array}$$

where *n* is the last value of a trial time series (see Figure 1C for exemplar LD time-series). Subsequently, *mean cumulative spatial error* (MCSE) based on the three CSE_trial_ values was computed. In addition, *movement time* (MT) and *functional range of motion* (F-ROM) were derived for each trial.

#### Evaluation of Proprioceptive Learning

We employed a psychophysical forced-choice paradigm to evaluate wrist proprioceptive acuity in both the PD and healthy control group. Participants wore opaque glasses and headphones playing pink noise to block visual and acoustic cues. In each trial, the robot passively moved the participants' wrist at a constant velocity of 6°/s to a 15°Flexion position (*standard stimulus*), held for 2 s, moved back to the starting position and then to another flexion position always >15° (*comparison stimulus*). Presentation order of the standard and comparison stimuli was randomized. At the end of each trial, participants verbally indicated the position farthest from the starting position (*first* or second position). Participant responses and stimulus positions were recorded. Based on the participant's response, the difference (*stimulus difference size*) between the standard and the comparison stimuli for the subsequent trial was increased/decreased using the *psi marginal method* (31, 32), an adaptive psychophysical algorithm. The procedure was repeated for 30 trials. Participants were allowed 1–2 min rest periods after 15 trials to ensure an active focus on the task. At the end of the assessment, a just-noticeable *discrimination threshold* (DT) was estimated by fitting correct response rates and stimulus difference sizes using a *logistic Weibull* function (31) for each participant.

#### Evaluating Local Motor Transfer

With vision and hearing masked as described as above, participants in both PD and healthy control performed previously untrained discrete wrist pointing movements. For a total of 20 trials, the WristBot passively moved the more affected wrist to a 15° target flexion position, held it for 2 s and moved back to the starting position. Then, participants actively moved their wrist to the previously perceived position. They could adjust their joint position until satisfied, then held the position for 2 s before the robot moved the joint to the starting position. Participants performed few familiarization trials before evaluation. For each participant, the mean absolute angular difference between target and joint position across trials was calculated as a *motor accuracy error* (MAE), representing the participant's ability to perform a goal-directed reaching movement based on proprioceptive information.

#### Evaluating Transfer to a Functional Handwriting Task

Participants in the PD group performed a battery of handwriting tests with their more affected hand using a Cintiq® Companion 2 tablet (Wacom Co., Ltd., Japan) paired with an active stylus for contact point recording. In a line-tracing task, participants traced the shape seen in the monitor at their own pace. A tracking task required participants to track the cursor moving in an arc, a straight line, or a spiral line. Arc and straight-line tasks required six back and forth movement repetitions at a rate of 0.5 Hz. First and last repetitions were not analyzed to control for beginning and end-of-the-movement inconsistencies. A spiral-line task required tracing or tracking a cursor moving in a concentric spiral line starting at the outer edge. Time-to-completion was recorded. Resultant root mean-squared error (RMSE) measures were derived based on horizontal and vertical position of the stylus contact position and the respective target tracing/tracking trajectory. These RMSE measures represented the ability to accurately move the stylus over the required trajectory either at a self-generated pace (tracing task) or at a required pace (tracking task).

## Results

### Task-Specific Motor Learning: Spatiotemoral Kinematics Improved During Training

**Figure 2:**
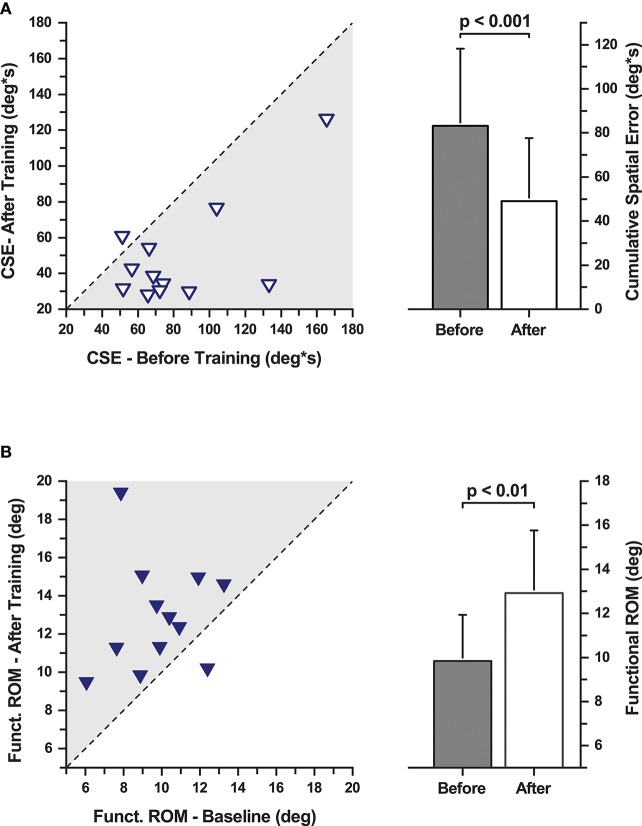
Cumulative spatial error (CSE) and functional range of motion during the evaluation of task-specific motor learning. (**A**, Left) CSE changes with training. Each data point represents a participant's CSE before and after training. The dashed line indicates the line of equality representing a no change in CSE as a function of training. (Right) Mean CSE across all participants before and after training. CSE decreased significantly after training. (**B**, Left) Functional range of motion changes with training. Each data point represents a participant's functional range of motion before and after training. The dashed line indicates the line of equality representing a no change as a function of training. (Right) Mean functional range of motion increased significantly after training.

Performance in the training task was evaluated in the PD group at baseline and after training (Figure 2). One PD participant could not complete all assessments resulting in an incomplete data set. Data of this participant were excluded from further analysis. The remaining 12 participants showed the following signs of motor learning: First, they significantly reduced the movement time, i.e., the time it took to successfully complete a trial (MT mean ± SD: 63.9 ± 37.7 s before training; 29.3 ± 18.2 s after training; effect size: *d* = 1.22 [very large]; repeated measures ANOVA: *F*_(1, 11)_ = 13.33, *p* = 0.004). Second, participants significantly reduced cumulative spatial error (MCSE mean ± SD: 83.08 ± 34.95 deg ⋅ s before training; 49.27 ± 28.65 deg ⋅ s after training; effect size: *d* = 1.10 [large]; repeated measures ANOVA: *F*_(1, 11)_ = 19.14, *p* = 0.001; Figure 2A). Third, functional range of motion during training increased significantly (F-ROM mean ± SD: 9.83° ± 2.11° before training; 12.93° ± 2.83° after training; effect size: *d* = −1.30 [very large]; repeated measures ANOVA: *F*_(1, 11)_ = 10.28, *p* = 0.008; Figure 2B). In summary, participants showed clear signs of training-related motor learning. With training, they completed the trials faster (−46%), reduced spatial error (−59%), and increased their functional range of motion when performing the task (+31.5%).

### Sensory Learning: Training Reduced Proprioceptive Discrimination Thresholds

**Figure 3:**
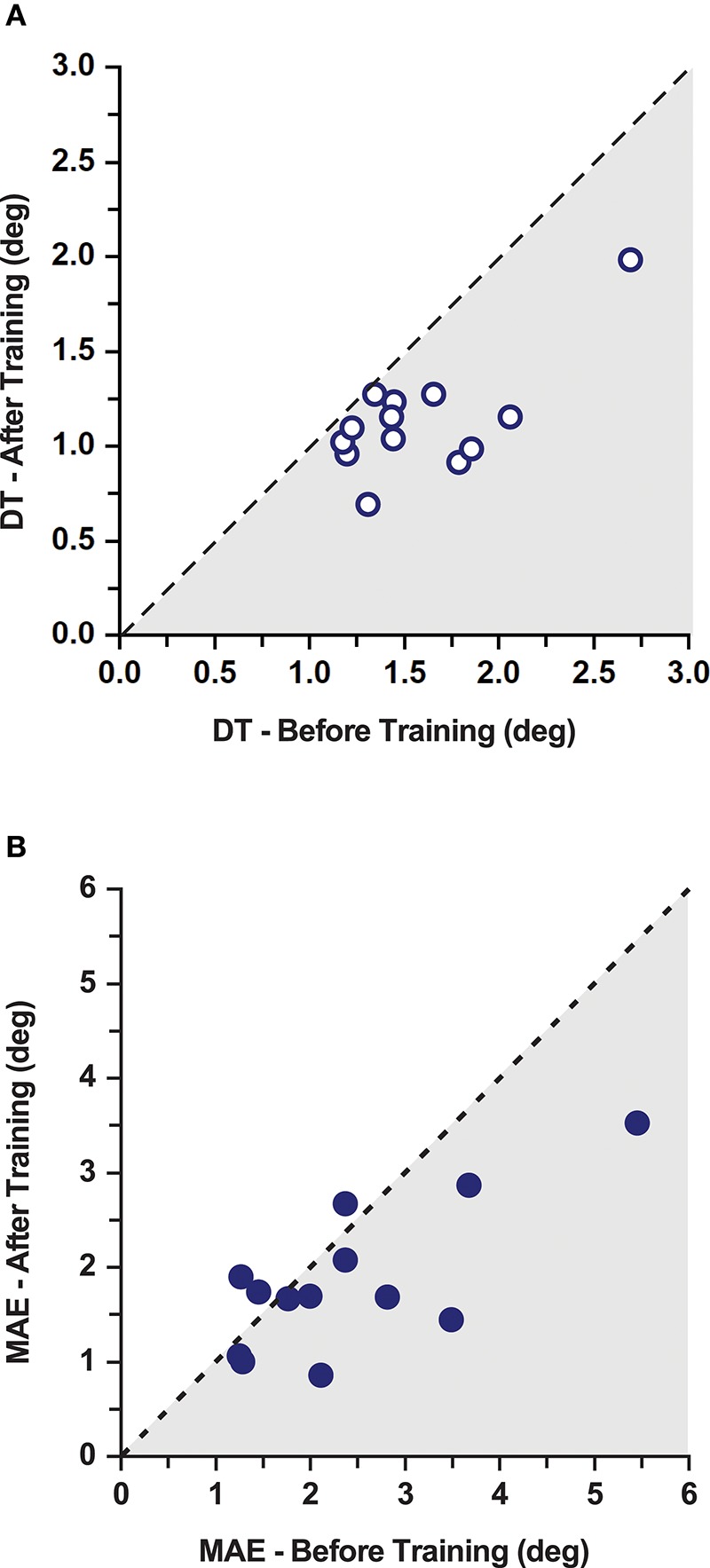
Effects of training on discrimination thresholds and movement accuracy in the PD gorup. **(A)** Proprioceptive discrimination thresholds (DT) before and after training. The dashed line indicates the line of equality representing a no change as a function of training. Shaded area marks the region of lower thresholds indicating improvements after training. **(B)** Movement Accuracy Error (MAE) before and after training. The dashed line indicates the line of equality representing a no change as a function of training. Shaded area marks the region of lower MAE indicating improvements after training. Note that 10/13 participants improved after training.

**Figure 4:**
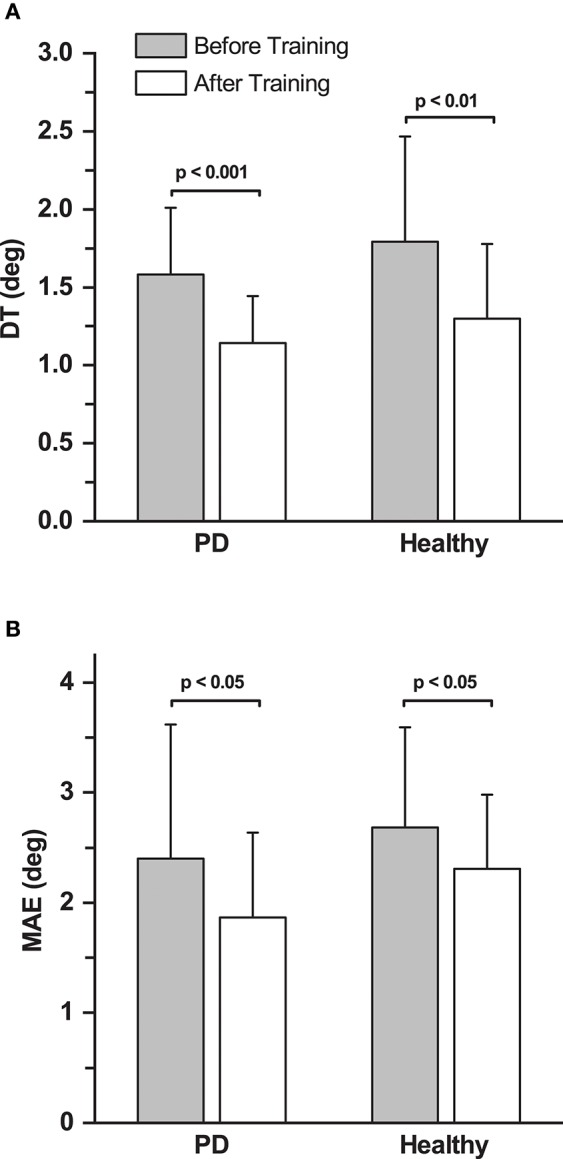
Effects of training on discrimination thresholds and movement accuracy. **(A)** Mean proprioceptive discrimination thresholds (DT) before and after training across all the participants in the PD and healthy control group. **(B)** Mean Movement Accuracy Error (MAE) before and after training across all the participants in both the PD and the healthy control group.

Double baseline discrimination thresholds (DT) were obtained for all PD participants. The two baseline DT means in the PD group were not significantly different from each other (DT mean ± SD: 1.61° ± 0.50° at *baseline 1*; and 1.56° ± 0.42° at *baseline 2*; repeated measures ANOVA: *F*_(1, 12)_ = 0.294, *p* = 0.598). Thus, both baseline data sets were collapsed into a single baseline DT for further analysis (combined mean ± SD: 1.58° ± 0.43°). In order to understand the training induced differences in the PD group, a repeated measures ANOVA was conducted between the mean DT at baseline and after training. Mean group DT after training was significantly lower than the combined baseline in the PD group (Mean ± SD: 1.14° ± 0.30°; 27.7% decrease; effect size: *d* = 1.24 [very large]; repeated measures ANOVA: *F*_(1, 12)_ = 26.49, *p* < 0.001; Figures 3A, 4A). A Pearson's product-moment correlation analysis contrasting the combined baseline DT against the absolute improvements in DT after training showed a strong positive correlation (*r* = 0.71, *p* = 0.006). This indicated that PD participants with higher baseline DT tended to achieve greater improvements in proprioceptive function, i.e., gaining greater benefits from training.

For the healthy control group, baseline DT (mean ± SD: 1.80° ± 0.65°) was assessed only once. Similar to the PD group, the control group showed significant improvements in DT after training (Mean ± SD: 1.32° ± 0.46°; 28.3% decrease; effect size: *d* = 0.89 [large]; repeated measures ANOVA: *F*_(1, 12)_ = 12.36, *p* < 0.01; Figure 4A). The DT at baseline and after training was not significantly different between the PD and the healthy control group (*p* > 0.05).

### Non-Task Specific Motor Learning: Motor Accuracy Error Was Reduced After Training

No systematic differences in movement accuracy for both baseline measures were found in the PD group (MAE mean ± SD: 2.50° ± 1.31° at *baseline 1*; 2.30° ± 1.17° at *baseline 2*; repeated measures ANOVA: *F*_(1, 12)_ = 1.96, *p* = 0.187). The average of the two baseline MAE measures for each participant served as baseline MAE (combined mean ± SD: 2.40° ± 1.21°). Ten out of 13 participants in the PD group showed improvements in MAE (Figure 3B). Mean MAE after training across all PD participants was 1.86° ± 0.77°, which was significantly different from the combined baseline (25.3% reduction in the 10 participants who improved; effect size: *d* = 0.55 [medium]; repeated measures ANOVA: *F*_(1, 12)_ = 5.38, *p* = 0.039; Figure 4B). The combined baseline MAE correlated strongly with absolute improvements in MAE after training (*r* = 0.78, *p* = 0.002), indicating that participant with higher baseline MAE tended to show largest improvements in movement accuracy after training. Baseline MAE measures across all participants in the healthy control group (mean ± SD: 2.67° ± 0.87°) did not differ significantly from the PD group (*p* > 0.05). Similar to the PD group, the healthy control group showed significant improvements in the MAE measures across the participants after training (Mean ± SD: 2.25° ± 0.67°; 13.9 % decrease; effect size: *d* = 0.56 [medium]; repeated measures ANOVA: *F*_(1, 12)_ = 7.82, *p* = 0.016; Figure 4B).

### Spatio-Temporal Measures in Functional Handwriting Did Not Improve

No systematic differences were found in RMSE values and time-to-completion in all the tracing and tracking tasks with training. Repeated measures ANOVA failed to identify any differences with training and yielded non-significant results in all the variables. Overall, participants did not show any systematic training related enhancements in both tracing and tracking tasks.

### Relationship Between Proprioceptive and Motor Learning

**Figure 5:**
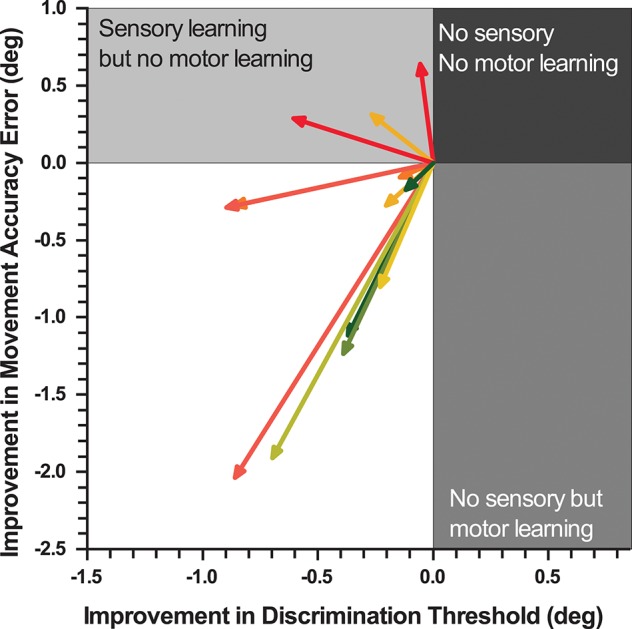
Vectorgram mapping gains in proprioceptive acuity against gains in movement accuracy. Each vector depicts a participant's sensorimotor gain. The coordinates of proprioceptive discrimination threshold and movement accuracy error score are aligned to the origin. The length of the vector indicates the magnitude of relative change and the angle the direction of change.

To contrast the relationship between the improvements in proprioceptive acuity and untrained motor performance, a Pearson's product-moment correlation analysis was performed between the absolute improvements in DT and MAE, which yielded a medium, but non-significant correlation (*r* = 0.46; *p* = 0.11). Figure 5 illustrates the improvements in both sensory and motor measures for each participant as a vector of the absolute improvement in MAE and DT. The representative mean vector angle across all participants was 36.7°From the negative X-axis, indicating that sensory and motor learning coincided and that participants achieved larger improvements in sensory than in motor measures (Rayleigh z_6.11_, *p* < 0.002).

### Effects of Disease Duration, Disease Severity, and Medication on Sensorimotor Outcome Measures

Disease duration correlated strongly with baseline DT (*r* = 0.77, *p* = 0.002) and moderately strong with MAE (i = 0.58, *p* = 0.05), indicating that wrist position sense and movement accuracy tended to decline with disease severity. Expectedly, disease duration correlated significantly with levodopa equivalence dosage (*r* = 0.73, *p* = 0.005). Age, gender, UPDRS motor score and levodopa equivalence dosage did not yield significant correlations with any of the sensory or motor outcome measures.

## Discussion

This study examined, if, and to what extent, proprioceptive function is trainable in PWPs. In addition, it determined the effects of such somatosensory-based training on motor function. Specifically, we investigated signs of a local transfer to an untrained motor behavior within the same joint degree-of-freedom, and a more global transfer to a functional handwriting task involving multiple degrees of freedom. The principal findings of this study are as follows: First, our robot-aided training significantly improved position sense in PD. Second, there was evidence for a local motor transfer within the same joint degree-of-freedom as movement accuracy in untrained non-visuomotor task improved with training. Lastly, there was no evidence for transfer of training to the multiple degree-of-freedom functional handwriting task. All PWPs improved motor performance in the trained visuomotor task. Given that the double baseline assessments of the motor and proprioceptive measures showed no differences, the above findings are not explainable as an effect of multiple testing.

### Training Results in Proprioceptive Improvements in PD

Our brief 30-min proprioceptive training induced improvements in wrist proprioceptive acuity in all PWPs. This finding provides first evidence that training can improve proprioceptive function in PD. The gain in proprioceptive learning in our sample of PD participants was considerable (28% reduction in threshold, see Figure 2), in comparison to the age-matched healthy controls in the study. In fact, the improvements in the PD group is similar to the gains achieved by young healthy adults (34%) who performed the identical training task (33), with the exception that young adults achieve these levels faster. Other studies on proprioceptive training in healthy adults [for a review see (18)] have also reported short-term training-related sensory change at approximately the same magnitude. This indicates that at least in the mild to moderate stages of PD, a somatosensory-based motor skill training can yield meaningful improvements in proprioceptive function at a level that is comparable to healthy adults. The neural mechanism behind these gains in proprioceptive function are likely processes of experience-dependent, short-term neural plasticity in somatosensory cortex that are active during behavioral training and are based on long-term potentiation and possibly depression (34).

Previous research in PD reported deficits on multisensory and sensorimotor integration. For example, altered visuo-proprioceptive integration has been documented by the characteristic misalignment of visually and proprioceptively perceived finger positions of PD participants (35), and the characteristic errors of undershooting movements when pointing to targets against gravity (36) or when learning the dynamics of an unknown force-field (37). The observable deficits in both visuo-proprioceptive as well as proprioceptive-motor integration have been attributed to the known proprioceptive dysfunction in PD [for reviews see (7, 8)]. That is, processes of multisensory integration, sensorimotor integration and adaptive motor learning that rely on proprioceptive inputs are all affected in PD. Our results document that proprioception is trainable in PD and is associated with positive outcomes in motor function. This, in turn, opens an avenue to explore, whether somatosensory-based forms of motor training may also improve the above processes of integration and adaptive motor learning.

### Training Generalizes to an Untrained Motor Task Within the Same Degree-Of-Freedom

Sensorimotor learning transferred to the untrained motor task within the same joint degree-of-freedom. By the end of practice, PD patients showed clear signs of motor learning. They exhibited an enlarged functional range of motion, which implies a “freeing” of this joint degree-of-freedom, a known sign of motor learning (38). In addition, they had reduced their movement time (−46%) and their spatial error (−59%) in the visuomotor, virtual ball-balancing task (see Figure 3A). By the end of practice, they also had significantly reduced spatial error (−31%) in the untrained pointing task, indicating that learning had transferred. The transferred learning gain in spatial error reduction was approximately half the gain of the practiced task, but closely resembled the gain in proprioceptive acuity (+28%). Thus, when considering motor accuracy measures the transfer was incomplete, but when considering the gain in position sense accuracy the transfer was nearly complete.

Whether the observable motor transfer was due to improvements in proprioceptive acuity or the result of motor learning, or a combination of both cannot be answered conclusively with this experiment. However, our data do indicate that somatosensory and motor learning coincided in the majority of the participants (77%) with those showing the largest improvements in position sense accuracy also exhibiting the largest gains in movement accuracy (see Figure 5).

### Training Does Not Generalize to Hand Writing Movements

Our training did not induce any measurable changes in the tracing and tracking task performance. These tasks involve multiple degrees of freedom at the hand and wrist and constitute a standardized handwriting evaluation with well-established sensitivity (39). Handwriting is a functional task that relies on the integration of visual and proprioceptive information and is classically affected in PD as manifested by “micrographia.” The fact that our training did not improve handwriting outcome measures implies that it was insufficient to induce global or functional motor transfer. There are at least two possible reasons for this negative outcome. First, the applied single degree-of-freedom training is too specific. Gains in the trained degree-of-freedom do not transfer to other degrees-of-freedom. Thus, tasks that involve multi-joint movements and joint rotations across multiple axes do not benefit from single-joint, single degree-of-freedom training. Second, the training dosage is insufficient. That is, a single brief training employed for about 30 min is just not enough to induce transfer effects. There is evidence from motor control studies in healthy adults to suggest that transfer within a trained degree-of-freedom is possible, but that such motor learning does not necessarily transfer to other degrees of freedom of the same joint (40), which is what we found in our sample of PD participants.

### Disease Duration Relates to Sensorimotor Performance

UPDRS motor scores and levodopa equivalent dosage did not correlate with any of the baseline or training related outcome measures, a finding consistent with previous studies (12, 13). We found that disease duration is associated with poorer proprioception and movement accuracy in PD, corroborating earlier findings (41, 42). Initial performance at baseline correlated strongly with training-related improvements in proprioceptive acuity (*r* = 0.71) and movement accuracy (*r* = 0.78). This implies that the more affected patients, who exhibited poorer motor performance and sensory acuity, showed the greatest learning gains. However, keep in mind that we tested only participants with mild to moderate PD. It is possible that PWP at later stages of the disease may no longer be able to realize the same learning gains.

## Conclusion

This is the first study to show successful proprioceptive learning in PD. These gains in proprioceptive acuity were associated with motor transfer. However, the transfer was local, restricted to the trained joint-degree-of-freedom. We observed no clear signs of a global transfer to a functional task involving the same degree-of-freedom. These findings provide a scientific basis for understanding why behavioral approaches that focus on body awareness, such as Tai Chi, Yoga or ball room dancing, can be successful adjuvant therapies for the symptomatic treatment of PD. Numerous other questions such as the retention of learning, optimal training duration and dosage to achieve maximal improvements, as well as the neural correlates of training remain and will need to be addressed in future research. Furthermore, the effect of proprioceptive training in various phenotypes of PD should be evaluated. This limited sample study provides a proof-of-concept that a somatosensory-based training can be beneficial in PD. A systematic clinical trial with a larger sample is needed to substantiate our initial findings on the effectiveness of this approach.

## Ethics Statement

The study procedure and consent process was performed with approval and under the accordance of relevant guidelines and regulations by Human Research Protection Program at the University of Minnesota.

## Author Contributions

NE, PT, and JK research project conception and design. NE acquisition of data. NE, PT, and JK analysis and interpretation of data. NE manuscript preparation. PT and JK manuscript review and critique.

### Conflict of interest statement

The authors declare that the research was conducted in the absence of any commercial or financial relationships that could be construed as a potential conflict of interest.
